# Fabrication of Large-Area Molybdenum Disulfide Device Arrays Using Graphene/Ti Contacts

**DOI:** 10.3390/molecules26154394

**Published:** 2021-07-21

**Authors:** Myungwoo Son, Jaewon Jang, Dong Chul Kim, Seunghyup Lee, Hyo-Soon Shin, Moon-Ho Ham, Sang-Soo Chee

**Affiliations:** 1 Artificial Intelligence and Energy Research Center, Korea Photonics Technology Institute (KOPTI), Gwangju 61007, Korea; mwson@kopti.re.kr; 2 Gwangju Institute of Science and Technology (GIST), School of Earth Sciences and Environmental Engineering (SESE), Gwangju 61005, Korea; jwjang@gm.gist.ac.kr; 3 Nanomaterials and Nanotechnology Center, Korea Institute of Ceramic Engineering and Technology (KICET), Jinju-si 52851, Korea; minamdc@kicet.re.kr (D.C.K.); shbelly@kicet.re.kr (S.L.); hshin@kicet.re.kr (H.-S.S.); 4 Gwangju Institute of Science and Technology (GIST), School of Materials Science and Engineering, Gwangju 61005, Korea; mhham@gist.ac.kr

**Keywords:** MoS_2_, graphene, interlayer, metal contacts, large-area

## Abstract

Two-dimensional (2D) molybdenum disulfide (MoS_2_) is the most mature material in 2D material fields owing to its relatively high mobility and scalability. Such noticeable properties enable it to realize practical electronic and optoelectronic applications. However, contact engineering for large-area MoS_2_ films has not yet been established, although contact property is directly associated to the device performance. Herein, we introduce graphene-interlayered Ti contacts (graphene/Ti) into large-area MoS_2_ device arrays using a wet-transfer method. We achieve MoS_2_ devices with superior electrical and photoelectrical properties using graphene/Ti contacts, with a field-effect mobility of 18.3 cm^2^/V∙s, on/off current ratio of 3 × 10^7^, responsivity of 850 A/W, and detectivity of 2 × 10^12^ Jones. This outstanding performance is attributable to a reduction in the Schottky barrier height of the resultant devices, which arises from the decreased work function of graphene induced by the charge transfer from Ti. Our research offers a direction toward large-scale electronic and optoelectronic applications based on 2D materials.

## 1. Introduction

Two-dimensional (2D) molybdenum disulfide (MoS_2_) has emerged as a post-silicon material because of its outstanding electrical and optical properties compared with its bulk counterparts [1,2]. In particular, its well-established scaled-up production and tunable bandgap depending on the number of layers make it promising for potential electronic and optoelectronic applications [1,2,3].

To realize electronic and optoelectronic devices based on MoS_2_, suitable contact engineering is essential because the Schottky barrier height (SBH) formed between MoS_2_ and metal contacts is critical for determining the field-effect mobility of the resultant device [4]. Various metal contact approaches have been implemented to improve the device performance over the last decade [4,5,6]. Das et al. used scandium contacts, an extremely low-work-function metal, to achieve an outstanding mobility (~700 cm^2^/V∙s) [4]. However, there was a significant difference between the experimental and theoretical SBHs extracted from the Schottky–Mott limit because the metal-induced Fermi level pinning effect prevents a shift in the Fermi level depending on the metal work function [7,8].

Several methods have been developed to overcome metal-induced Fermi level pinning, including interlayer and phase engineering [9,10]. In particular, a graphene interlayer can suppress metal-induced Fermi level pinning by blocking the interaction between the metal and MoS_2_ [9,11]. Moreover, the interface between graphene and 2D materials is sharp and clean, which leads to efficient charge transfer [12]. Owing to such positive effects, there have been a few reports on graphene-interlayered MoS_2_ devices [9,11,13], leading to improvements in their electrical properties compared with conventional metal contacts [9,11]. However, graphene has a higher work function than MoS_2_ [14,15], implying that the SBH is even higher than that of conventional low-work-function metal contacts. Furthermore, mechanically exfoliated MoS_2_ flakes have been commonly utilized, which cannot be applied in large-scale devices because of their limited flake size [9,11]. Therefore, it is highly desirable to develop interlayer engineering that enables practical large-scale electronic and optoelectronic applications while achieving a high performance.

In this study, we developed graphene-interlayered Ti contacts (graphene/Ti contacts) to improve the device performance of large-area MoS_2_ films grown by chemical vapor deposition (CVD). Ti is a low-work-function metal [16], which can overcome the high work function of graphene because of the efficient Fermi level tunability afforded by metal deposition. To confirm the effects of graphene/Ti contacts, we also fabricate conventional Ti-contacted MoS_2_ devices and characterize their electrical and photoelectrical properties.

## 2. Results and Discussion

**Figure 1:**
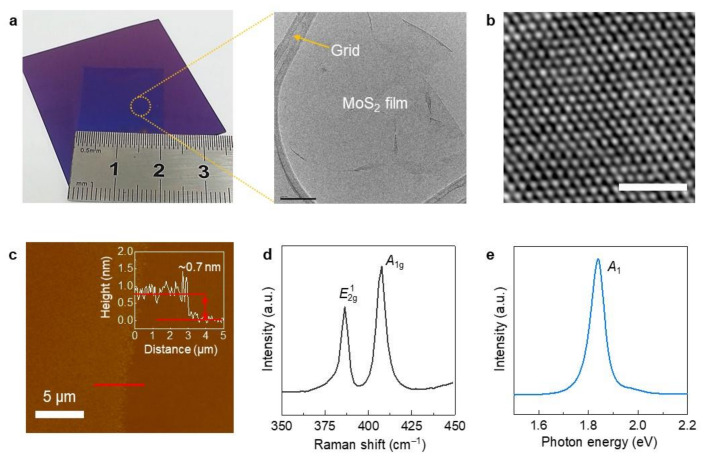
Characterization of MoS_2_ monolayer film grown by CVD: (**a**) digital micrograph and TEM images (scale bar denotes 100 nm), (**b**) high-resolution STEM image (scale bar denotes 1 nm), (**c**) AFM image (inset: line profile graph marked by red line), (**d**) Raman spectrum, and (**e**) PL spectrum.

Figure 1a shows the MoS_2_ film synthesized on a Si/SiO_2_ substrate. Overall, the synthesized MoS_2_ film shows a good uniformity with no noticeable cracks (Figure 1a). High-resolution scanning transmission electron microscopy (STEM) shows a three-fold coordinated lattice structure [17]. A thickness of 0.7 nm was measured by atomic force microscopy (AFM) analysis, which implies that our MoS_2_ film is a monolayer (Figure 1b,c) [18]. The Raman spectrum of the MoS_2_ film shows two main peaks corresponding to $E_{2g}^{1}$ and *A*_1g_ originating from in-plane and out-of-plane vibrations [18,19], respectively (Figure 1d). From this spectrum, we estimate the peak difference (*A*_1g_ − $E_{2g}^{1}$) and the full-width at half-maximum (FWHM) of the $E_{2g}^{1}$ peak, which are associated with the number of layers and film quality, respectively [18,19]. The peak difference and FWHM were estimated to be approximately 20 and 4.1 cm^−1^, respectively, which reveal that our MoS_2_ film is a monolayer and that its quality is comparable to that obtained by mechanical exfoliation [18,19].

The photoluminescence (PL) spectrum was also obtained to confirm the exciton energy of the MoS_2_ film (Figure 1e). A strong peak was observed at 1.84 eV (*A*_1_), which further confirms that our MoS_2_ film is a monolayer [19]. This PL analysis is well matched with the STEM, Raman, and AFM analyses.

**Figure 2:**
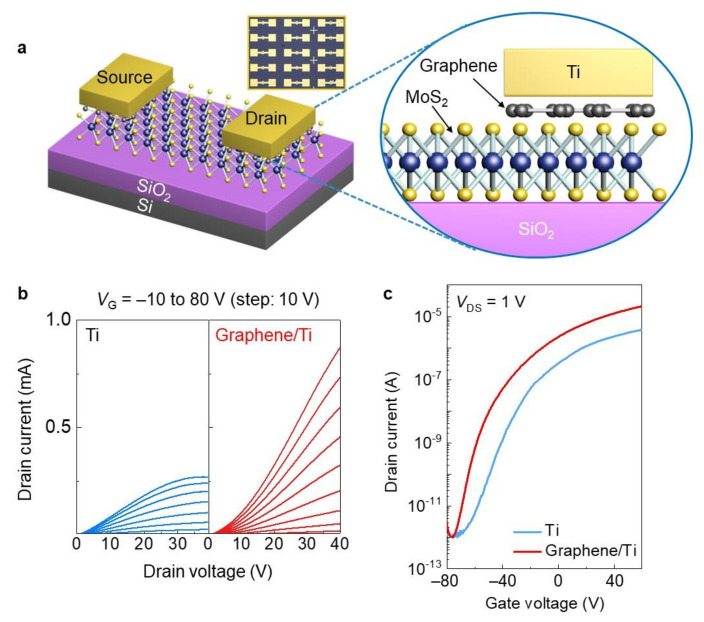
(**a**) Schematic illustration of graphene/Ti-contacted MoS_2_ device (inset: optical microscopy image of MoS_2_ device arrays with graphene/Ti contacts). (**b**) Output and (**c**) transfer characterizations of MoS_2_ devices with Ti and graphene/Ti contacts.

Figure 2a shows a schematic illustration of the MoS_2_ devices with graphene/Ti contacts. The device fabrication step was inspired from Chee et al. [13]. We first synthesized a graphene monolayer film grown by CVD (Figure S1), following a wet transfer on Si/SiO_2_ substrate. After that, we fabricated the patterned graphene using a standard photolithography. Once graphene was patterned, we transferred graphene patterns onto CVD-grown MoS_2_ film using a wet transfer method again. We then patterned the source and drain electrodes on top of the graphene. Finally, we directly deposited 50 nm-thick Ti and then 10 nm-thick Au to prevent the oxidation of Ti. Contrary to methods in previous reports on graphene interlayer-contacted devices, our process enables the realization of large-area MoS_2_ device arrays (inset in Figure 2a). We explored the electrical properties of the MoS_2_ devices with Ti and graphene/Ti contacts to confirm the effect of the graphene interlayer. As shown in Figure 2b,c, the MoS_2_ devices exhibit identical *n*-type behaviors regardless of the type of metal contact. Interestingly, graphene/Ti contacted devices present more linear-dominant behavior, and the on-state current level is also significantly enhanced, compared with the Ti contacted devices. Thanks to this improvement, the device performances of the MoS_2_ device with graphene/Ti contacts are also improved (mobility: 18.3 cm^2^/V∙s, on/off current ratio: 3.1 × 10^7^) relative to the device with Ti contacts (mobility 3.2 cm^2^/V∙s, on/off current ratio: 4.5 × 10^6^). In general, the enhancement in the field-effect mobility is generally accompanied with a reduction in the on/off current ratio [20], while our device with graphene/Ti contacts presents an identical off-state current level, compared with conventional Ti contacted devices.

This outstanding improvement is attributed to the reduced SBHs of the resultant devices using the graphene/Ti contacts, leading to efficient charge transfer from the graphene/Ti contacts to the MoS_2_ channel [13,21].

**Figure 3:**
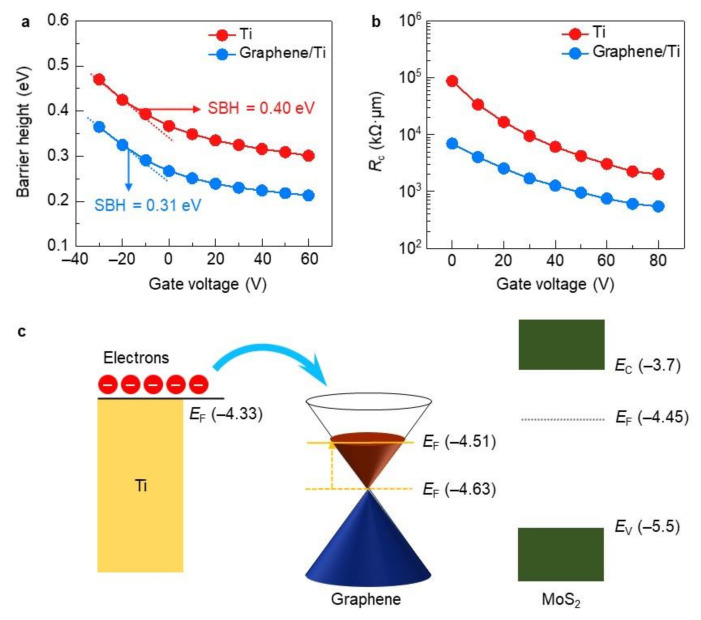
(**a**) Barrier height and (**b**) contact resistance (*R*_c_) of MoS_2_ devices with Ti and graphene/Ti contacts. Barrier heights were obtained from the data in Figure S2. (**c**) Band alignment of MoS_2_ monolayer contacted with graphene/Ti, where the Fermi levels (*E*_F_) of graphene and MoS_2_ were extracted from the data in Figure S3.

To prove our hypothesis, we quantitatively estimate the SBHs using a modified Richardson plot inspired by Tataroğlu et al. [22]:

(1) $$\ln\left(\frac{I_{0}}{T^{2}}\right)-\left(\frac{q^{2}\sigma^{2}}{2k^{2}T^{2}}\right)=\ln\left(AA^{*}\right)-\frac{q\varphi_{B}}{kT}$$

where *I*_0_ is the saturation current level, *T* is the temperature (K), *q* is the electronic charge, *k* is the Boltzmann constant, *σ* is the standard deviation of the Gaussian function of the SBH, *A* is the device area, A* is the Richardson constant, and *φ*_B_ is the barrier height. The Richardson constant is strongly related to the effective mass of the material and can be defined as A* = 4πqm*k^2^/*h*^3^, where m* is the effective mass of MoS_2_ and *h* is Planck’s constant [22,23]. From these equations, we plotted ln(*I*_0_/*T*^2^) − [(*q*^2^*σ*^2^)/(2*k*^2^*T*^2^)] of the MoS_2_ devices with Ti and graphene/Ti contacts as a function of *q*/(*kT*) (Figure S2). Using these graphs, the SBHs for each MoS_2_ device can be extracted as a function of the gate voltage by linear fitting under flat-band gate voltage conditions (Figure 3a), which corresponds to a point of deviation from the linear fit [4,13]. The extracted SBHs of the devices with Ti and graphene/Ti contacts were 0.40 and 0.31 eV, respectively. The reduced SBH with the graphene/Ti contacts was accompanied by a reduction in the contact resistance (*R*_C_) with respect to the device with the Ti contacts, which is a result of an improvement in the electrical properties (Figure 2c and Figure 3b).

This result correlates with output and transfer characterizations (Figure 2b,c). Wang et al. also demonstrated that the use of an interlayer can enhance the electrical properties of MoS_2_ devices by reducing the SBH [24].

To unveil why the SBH is reduced with the use of the graphene/Ti contacts in detail, ultraviolet photoelectron spectroscopy (UPS) measurements were carried out (Figure 3c and Figure S3). The work function of pristine graphene is approximately 4.63 eV, which is higher than that of MoS_2_ (4.45 eV). This means that a higher SBH would be formed between pristine graphene and MoS_2_. In contrast, the work function of graphene on a Ti film appears to be 4.51 eV, which implies that Ti induces an *n*-type doping effect on graphene [13,25], and consequently, the SBH can be lowered when graphene/Ti is in contact with MoS_2_ (Figure 3a,c). Considering that the lower work function metal is utilized rather than Ti, the SBH would be further reduced, and the electrical properties would also be enhanced. This result can be also supported by a transfer curve of an only graphene-contacted device, but not an interlayer contacted one (Figure S4). Its device performance is also improved (mobility: 7.8 cm^2^/Vs) compared with Ti contacted devices, but the contact effect is weaker than the graphene/Ti contacted device. This suggests that the coupling with graphene and low work function metals (Ti), is significantly beneficial to the MoS_2_ device performance. 

**Figure 4:**
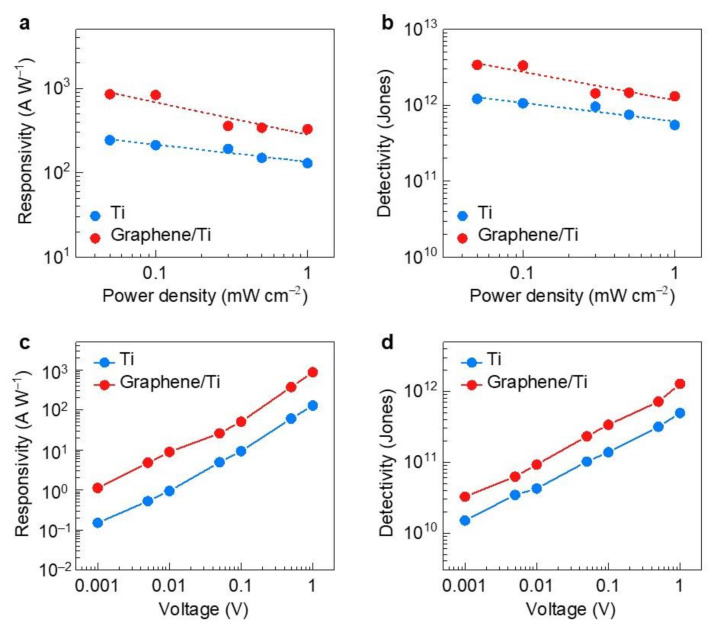
(**a**) Responsivities and (**b**) detectivities of MoS_2_ devices with Ti and graphene/Ti contacts under an applied bias of 1 V and V_G_ = 0 V and light illumination at 470 nm as a function of light power density. (**c**) Responsivities and (**d**) detectivities of MoS_2_ devices with Ti and graphene/Ti contacts under light illumination at 470 nm as a function of an applied bias, under V_G_ = 0 V. All graphs were acquired from the data in Figure S5.

Next, we characterized the photoelectrical properties of the MoS_2_ devices depending on the metal contacts under light illumination at 470 nm (Figure 4). Both the Ti- and graphene/Ti-contacted devices showed increased photocurrent levels under illumination, but the increase was larger for the graphene/Ti-contacted device. This is attributed to the boosted mobility resulting from the reduced SBH [13]. To quantify the photoelectrical performance, we estimated the power-dependent responsivity (*R*), defined as *R* = *I*_ph_/(*AP*), where *I*_ph_ is the photocurrent, *A* is the device area, and *P* is the light power density (Figure 4a).

For both devices, the responsivity decreases linearly with increasing power density. This is associated with an increase in scattering events between photogenerated carriers due to the high-power density [13,26], and a behavior that has been commonly demonstrated in TMDC photodetector devices. We note that the maximal responsivity of the device with graphene/Ti contacts is approximately 850 A/W, which is significantly higher (by a factor of 3.5) than that of the Ti-contacted device. This value is also considerably higher than previously reported values from studies on the performance of TMDC photodetectors [27,28].

The detectivity (D*) is another figure of merit for the sensitivity of photodetectors, which can be expressed as D* = *RA*^1/2^/(2*qI*_dark_)^1/2^, where *R* is the responsivity, *A* is the device area, *q* is the electronic charge, and *I*_dark_ is the dark current. A detectivity of ~2 × 10^12^ Jones is achieved for the device with graphene/Ti contacts, which is higher than that of the Ti-contacted devices (Figure 4b). Lastly, we characterized the responsivities and detectivities as a function of the applied bias (Figure 4c,d). With an increase in the applied bias, the responsivities and detectivities increased linearly for both contacted devices, but the values of graphene/Ti contacted devices were higher, with respect to Ti contacted one. This means that the outstanding photoelectrical performance of the graphene/Ti-contacted device at even an extremely low bias can be attributed to an improved mobility and low recombination rate between photogenerated carriers [29].

## 3. Materials and Methods

### 3.1. Synthesis of MoS_2_ and Graphene Monolayer Films

The MoS_2_ film was synthesized by CVD using both powders, MoO_3_ (99.999%, Materion Advanced Chemicals, Seoul, Korea) and sulfur (99.9% Sigma Aldrich, Saint Louis, MO, USA). Each powder was loaded into two separate alumina crucibles; the MoO_3_ crucible was positioned at the center of a quartz tube, and the sulfur crucible was placed upstream of the MoO_3_ crucible. A Si/SiO_2_ substrate was placed downstream of the MoO_3_ crucible. The tube furnace was heated to 650 °C and then kept for 40 min under 50 sccm of Ar carrier gas at 300 mTorr. After that, the tube furnace was naturally cooled to room temperature with 200 sccm of Ar gas. A graphene film was synthesized on a Cu catalyst (Alfa Aesar, Ward Hill, MA, USA) using CVD by a process similar to the MoS_2_ synthesis. The detailed synthesis procedure is described in our previous report [30].

### 3.2. Fabrication of MoS_2_ Devices with Ti and Graphene/Ti Contacts

MoS_2_ devices were fabricated using a standard photolithography method. First, MoS_2_ films were synthesized on a heavily *p*-doped Si substrate with a 300 nm-thick SiO_2_ layer, which acted as the bottom gate and dielectric layer, respectively. To prepare the patterned graphene, we used standard photolithography and an oxygen plasma etching system. Once graphene was patterned, the graphene electrodes were transferred onto the MoS_2_ film using a wet-transfer method, where a buffered oxide etch solution was used to etch the SiO_2_ layer. Then, secondary photolithography was performed on the graphene electrodes, followed by metal deposition (50 nm-thick Ti and then 10 nm-thick Au). Channel definition was conducted with a length of 14 μm and width of 40 μm using a third photolithography step, and then an unprotected MoS_2_ area was etched using Ar plasma treatment. For comparison, a MoS_2_ device with Ti contacts was prepared by identical processes except for preparation of the graphene electrodes.

### 3.3. Characterization

We performed optical microscopy (BX51, Olympus, Seoul, Korea), AFM (XE-100, Park Systems, Suwon, Korea), and Raman and PL spectroscopy (LabRAM HR Evolution, Horiba Jovin-Yvon, Kyoto, Japan) measurements to investigate the physical properties of the MoS_2_ films grown by CVD. To explore the work functions of the MoS_2_ and graphene films, UPS (Riken Keiki instrument, AC-2, Tokyo, Japan) measurements were carried out. To characterize the electrical and photoelectrical properties of the resultant devices, we used a semiconducting parameter analyzer (E5270B, Agilent Technologies, Santa Clara, CA, USA) under vacuum (10^−5^ mbar) at 80–300 K and a visible light lamp (470 nm).

## 4. Conclusions

In conclusion, we fabricated CVD-grown MoS_2_ device arrays with graphene/Ti contacts via a wet-transfer method. Using the graphene/Ti contacts, we achieved remarkable electrical and photoelectrical performances compared with the Ti-contacted devices, including a field-effect mobility of 18.3 cm^2^/V∙s, on/off current ratio of 3.1 × 10^7^, responsivity of 850 A/W, and detectivity of 2 × 10^12^ Jones. Such enhancements can be explained by the reduced SBHs coming from a decrease in the work function of the graphene interlayer induced by electron charge transfer from the low-work-function Ti metal. This interaction is more beneficial to boost device performances, compared with only graphene-contacted devices. Our contact engineering enables easy expansion of the doping range of graphene using various metal contacts, and directly combines high-k dielectric layers [31], achieving the high-performance 2D devices. This also facilitates realization of the 2D TMDC-based photodiode [32], thanks to the Fermi level de-pinning effect. Therefore, our findings provide insight for realizing practical large-scale electronic and optoelectronic applications based on 2D TMDCs.

## Data Availability

The data presented in this study are available on request from the corresponding author.

## Supplementary Materials

Figure S1: Characterization of graphene film, Figure S2: ln(*I*_0_/*T*^2^) − (*q*^2^σ^2^/2*K*^2^*T*^2^) vs. *q*/*KT* plots depending on contact types, Figure S3: UPS results of MoS_2_, Ti and graphene/Ti films, Figure S4: transfer characterizations of MoS_2_ devices with Ti, graphene, and graphene/Ti contacts, Figure S5: power-dependent *I*-*V* curves of MoS_2_ devices with Ti and graphene/Ti contacts.

## Sample Availability

Samples of the compounds are not available from the authors.
