# Supplementary material for: Fabrication of Large-Area Molybdenum Disulfide Device Arrays Using Graphene/Ti Contacts
Source: Molecules. 2021 Jul 21;26(15):4394. doi: 10.3390/molecules26154394 (PMC8348625; doi:10.3390/molecules26154394)
Supplement: Supplementary file 1 [file molecules-26-04394-s001.zip › molecules-1280083-supplementary.pdf]

*Supplementary Materials*

# Fabrication of Large-Area Molybdenum Disulfide Device Arrays using Graphene/Ti Contacts

Myungwoo Son [1,†], Jaewon Jang [2,†], Dong Chul Kim [3], Seunghyup Lee [3], Hyo-Soon Shin [3], Moon-Ho Ham [4], and Sang-Soo Chee [3,*]

[†] These authors contributed equally to this work.

[1]Artificial Intelligence and Energy Research Center, Korea Photonics Technology Institute (KOPTI), Gwangju 61007, Republic of Korea

[2]School of Earth Sciences and Environmental Engineering, Gwangju Institute of Science and Technology (GIST), Gwangju, 61005, Republic of Korea

[3]Nanomaterials and Nanotechnology Center, Korea Institute of Ceramic Engineering and Technology (KICET), Jinju-si, Gyeongsangnam-do 52851, Republic of Korea

[4]School of Materials Science and Engineering, Gwangju Institute of Science and Technology (GIST), Gwangju, 61005, Republic of Korea

*Correspondence: sschee@kicet.re.kr (S.S.C.)

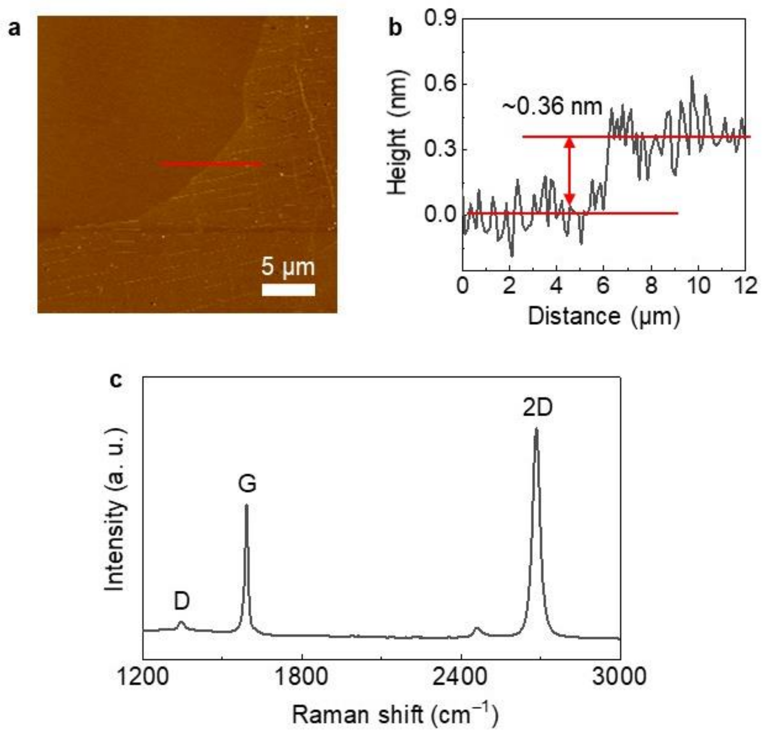


**Figure S1.** (a) AFM image, (b) line profile graph extracted from the red line in Fig. S1a, and (c) Raman spectrum of CVD-grown graphene film on a $Si/SiO_2$ substrate.

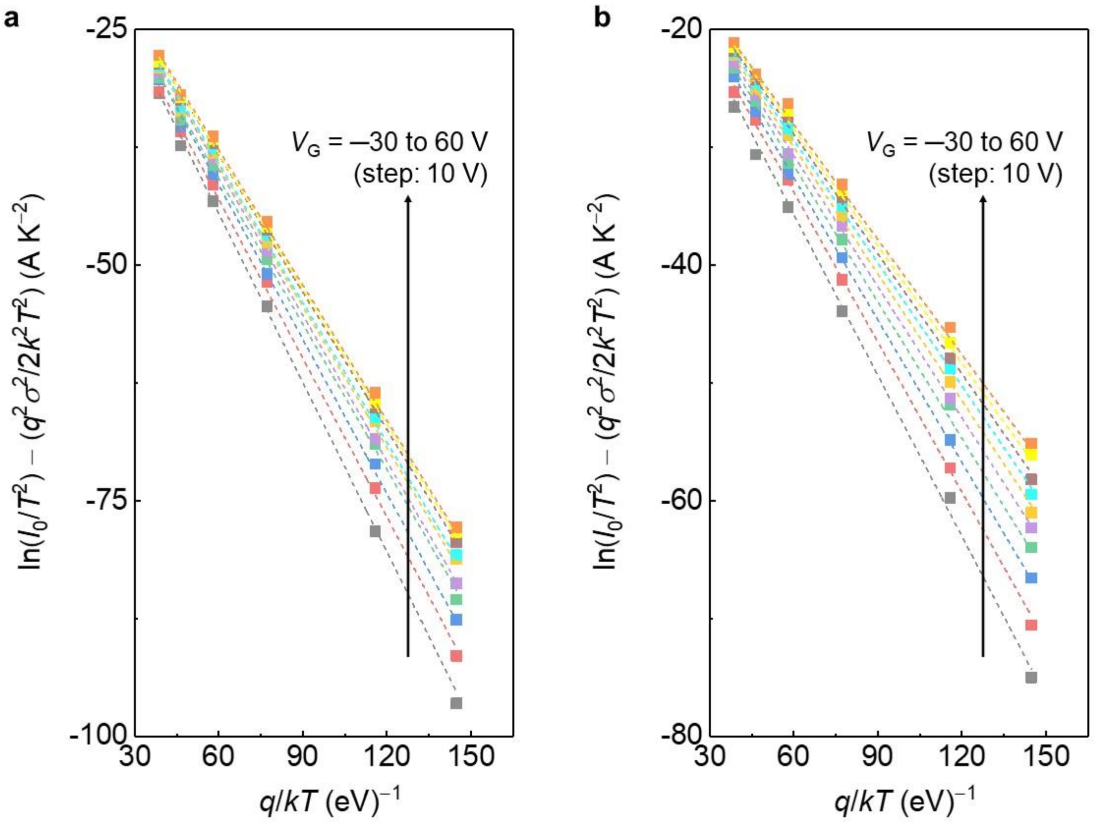


**Figure S2.** $\ln(I_0/T^2)–(q^2\sigma^2/2k^2T^2)$ vs. $q/kT$ plots of $MoS_2$ devices with (a) Ti and (b) graphene/Ti contacts, as a function of the gate voltage.

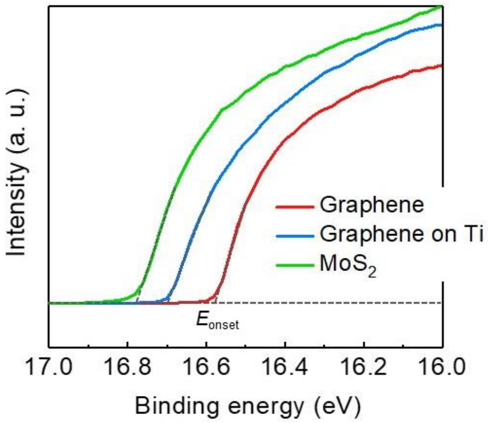


**Figure S3.** (a) UPS spectra of CVD-grown graphene (red), graphene on Ti substrate (blue), and CVD-grown $MoS_2$ films (green).

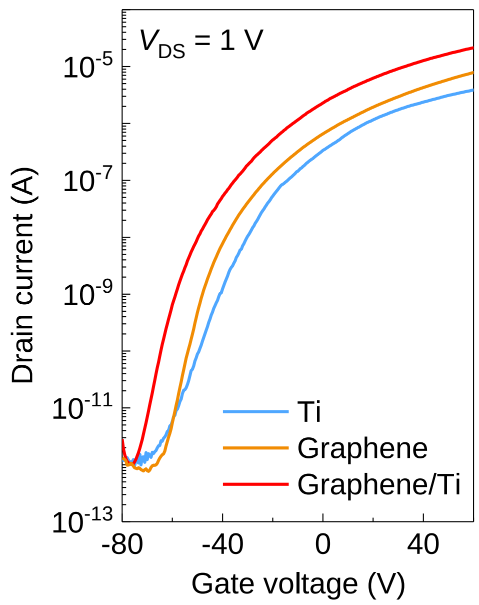


**Figure S4.** Transfer characterizations of $MoS_2$ devices with Ti, graphene, and graphene/Ti contacts. The data of Ti and graphene/Ti-contacted devices are taken from Fig. 2c.

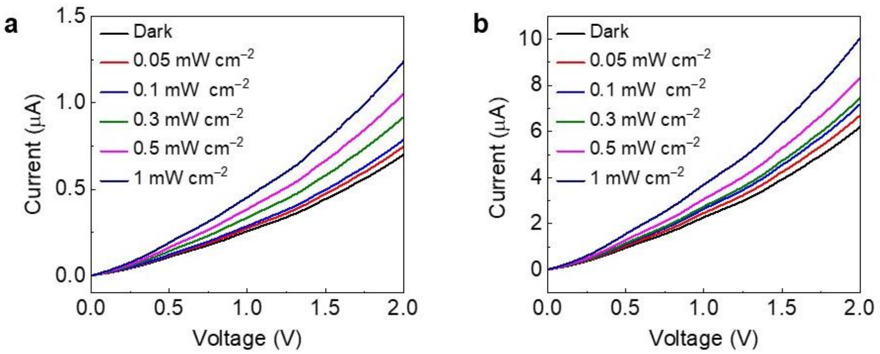


**Figure S5.** Power dependent *I-V* curves of $MoS_2$ devices with (a) Ti and (b) graphene/Ti contacts in the dark and under 470 nm light illumination.
